# First thermostable CLIP-*tag* by rational design applied to an archaeal *O^6^*-alkyl-guanine-DNA-alkyl-transferase

**DOI:** 10.1016/j.csbj.2022.09.015

**Published:** 2022-09-18

**Authors:** Rosa Merlo, Rosanna Mattossovich, Marianna Genta, Anna Valenti, Giovanni Di Mauro, Alberto Minassi, Riccardo Miggiano, Giuseppe Perugino

**Affiliations:** a Institute of Biosciences and BioResources, National Research Council of Italy, Via Pietro Castellino 111, 80131 Naples, Italy; b Department of Pharmaceutical Sciences, University of Piemonte Orientale, Via Bovio 6, 28100 Novara, Italy; c Department of Biology, University of Naples “Federico II”, Complesso Universitario di Monte S. Angelo, Ed. 7, Via Cinthia 26, 80126 Naples, Italy

**Keywords:** AGT, OGT, MGMT, *O^6^*-alkyl-guanine-DNA-alkyl-transferase, BC, *O^2^*-benzyl-cytosine, BG, *O^6^*-benzyl-guanine, HTH, helix-turn-helix motif, IMAC, immobilized metal affinity chromatography, SLP, Self-Labelling *Protein-tag*, *Protein-tag*, Protein labelling, Orthogonal substrate specificity, Thermozymes, Protein engineering

## Abstract

Self-labelling protein tags (SLPs) are resourceful tools that revolutionized sensor imaging, having the versatile ability of being genetically fused with any protein of interest and undergoing activation with alternative probes specifically designed for each variant (namely, SNAP-*tag*, CLIP-*tag* and *Halo-tag*). Commercially available SLPs are highly useful in studying molecular aspects of mesophilic organisms, while they fail in characterizing model organisms that thrive in harsh conditions. By applying an integrated computational and structural approach, we designed a engineered variant of the alkylguanine-DNA-alkyl-transferase (OGT) from the hyper-thermophilic archaeon *Saccharolobus solfataricus* (*Ss*OGT), with no DNA-binding activity, able to covalently react with *O^6^*-benzyl-cytosine (BC-) derivatives, obtaining the first thermostable CLIP-*tag*, named *Ss*OGT-*MC^8^*.

The presented construct is able to recognize and to covalently bind BC- substrates with a marked specificity, displaying a very low activity on orthogonal benzyl-guanine (BG-) substrate and showing a remarkable thermal stability that broadens the applicability of SLPs. The rational mutagenesis that, starting from *Ss*OGT, led to the production of *Ss*OGT-*MC^8^* was first evaluated by structural predictions to precisely design the chimeric construct, by mutating specific residues involved in protein stability and substrate recognition. The final construct was further validated by biochemical characterization and X-ray crystallography, allowing us to present here the first structural model of a CLIP-*tag* establishing the molecular determinants of its activity, as well as proposing a general approach for the rational engineering of any *O^6^*-alkylguanine-DNA-alkyl-transferase turning it into a SNAP- and a CLIP-*tag* variant.

## Graphical abstract

*Lessons from molecular evolution*: the acquired knowledge on the human MGMT is the starting point for applying a rational design to other AGTs, in order to obtain alternative (in our case thermostable) homologues and expand the “SNAP-*tag* technology” to organisms and/or reaction conditions incompatible with the activity of the commercial protein-tags.

**Figure:**
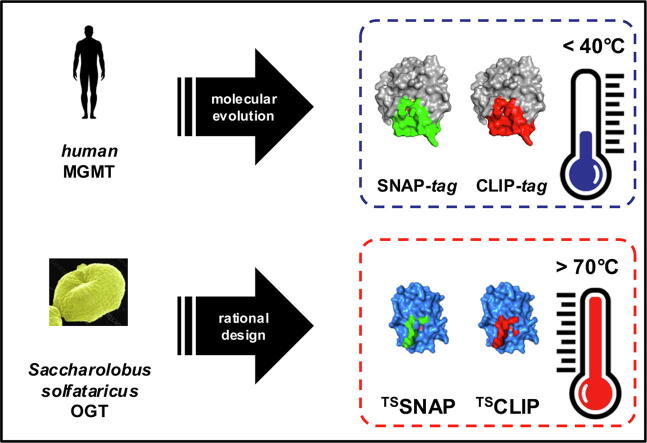

## 1 Introduction

**Fig. 1:**
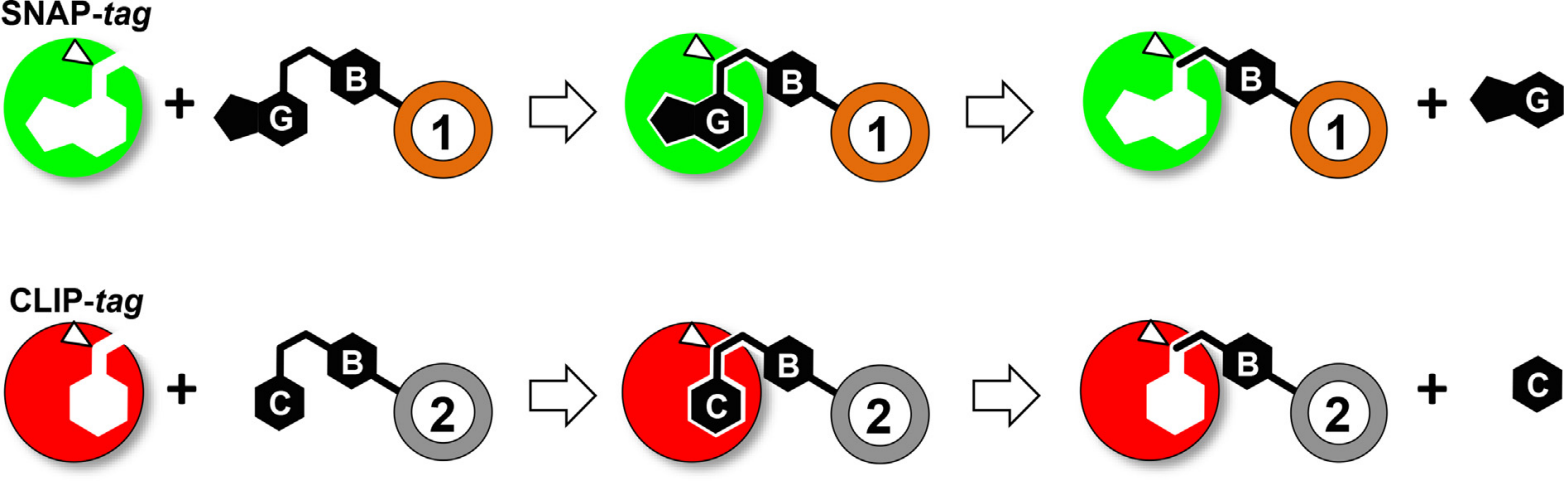
*Scheme of the irreversible reaction of SNAP- and CLIP-tag on their respective BG- and BC-derivative substrates*. Upon the reaction, the benzyl moiety is covalently linked to the catalytic cysteine (*white triangle*). If desired chemical groups (indicated as 1 and 2) are previously conjugated to BG- and BC-, it is possible to achieve a specific multi-protein labelling by these commercial SLPs.

*Self-labelling protein-tags* (SLPs) catalyse the covalent, highly specific and irreversible binding of the fluorogenic moiety of synthetic ligands designed by mimicking the physiological protein substrate. Their relatively small dimension makes them powerful tools for a huge number of applications [1]. To this aim, in 2003, the group of Prof. Kai Johnsson introduced for the first time an engineered version of the human *O^6^*-alkyl-guanine-DNA-alkyl-transferase (hMGMT), by applying a molecular evolution approach in order to: *i*), erase the protein DNA binding activity, *ii*) increase the catalytic activity toward the pseudo-substrate *O^6^*-benzyl-guanine (BG-) derivatives [2], [3], [4], [5], [6] (Fig. 1). The unique features of this protein that covalently retain a part of the BG- substrate allowed a huge number “biotech” applications: conceptually, all desired chemical groups, if previously conjugated to the benzyl moiety, could be covalently linked to the protein [2], [3], [4], [5], [6]. Accordingly, an impressive number of papers appeared in literature, leading to the commercialization of this engineered hMGMT as “SNAP-*tag*” (New England Biolabs) whose application cover various disciplines, from medicine to biotechnology: genetically fused to a protein of interest (POI), it has been proposed as valid alternative to fluorescent proteins (FPs) and *affinity-tags proteins* (as GST, MBP, His-*tag*, etc.) mainly in fluorescence microscopy [7], but also in protein purification/immobilization methodologies [8]. Noteworthy, despite the disadvantage to utilise an external substrate, SNAP-*tag* revealed particularly useful in some contexts, as anaerobic conditions, where FPs fail to produce a fluorescent signal [9]. Furthermore, the high enzyme specificity for the substrate and the extreme versatility of the production of BG-conjugates make the SNAP-*tag* an ideal tool for the production and development of new generation biosensors [10], [11].

Afterwards, the need to get an available SLP with an orthogonal substrate specificity with respect to the SNAP-*tag* for *in vivo* multi-protein labelling, induced Johnsson and co-workers to a further development, again by molecular evolution, of a variant of the hMGMT (the CLIP-*tag*), able to recognise *O^2^*-benzyl-cytosine (BC-) derivatives [12] (Fig. 1). This is peculiar, because the great majority of model organisms does not react with BC-, avoiding any endogenous activity [12]. The advantage of possessing two orthogonal SLPs to be genetically fused to two respective POIs is particularly suitable for *in vivo* and *in vitro* protein–protein interaction studies, by methodologies as the Selective crosslinking of interacting proteins (S-CROSS) [13], as well as by employing FRET fluorophore pairs conjugated to the BG- and BG- substrates.

**Fig. 2:**
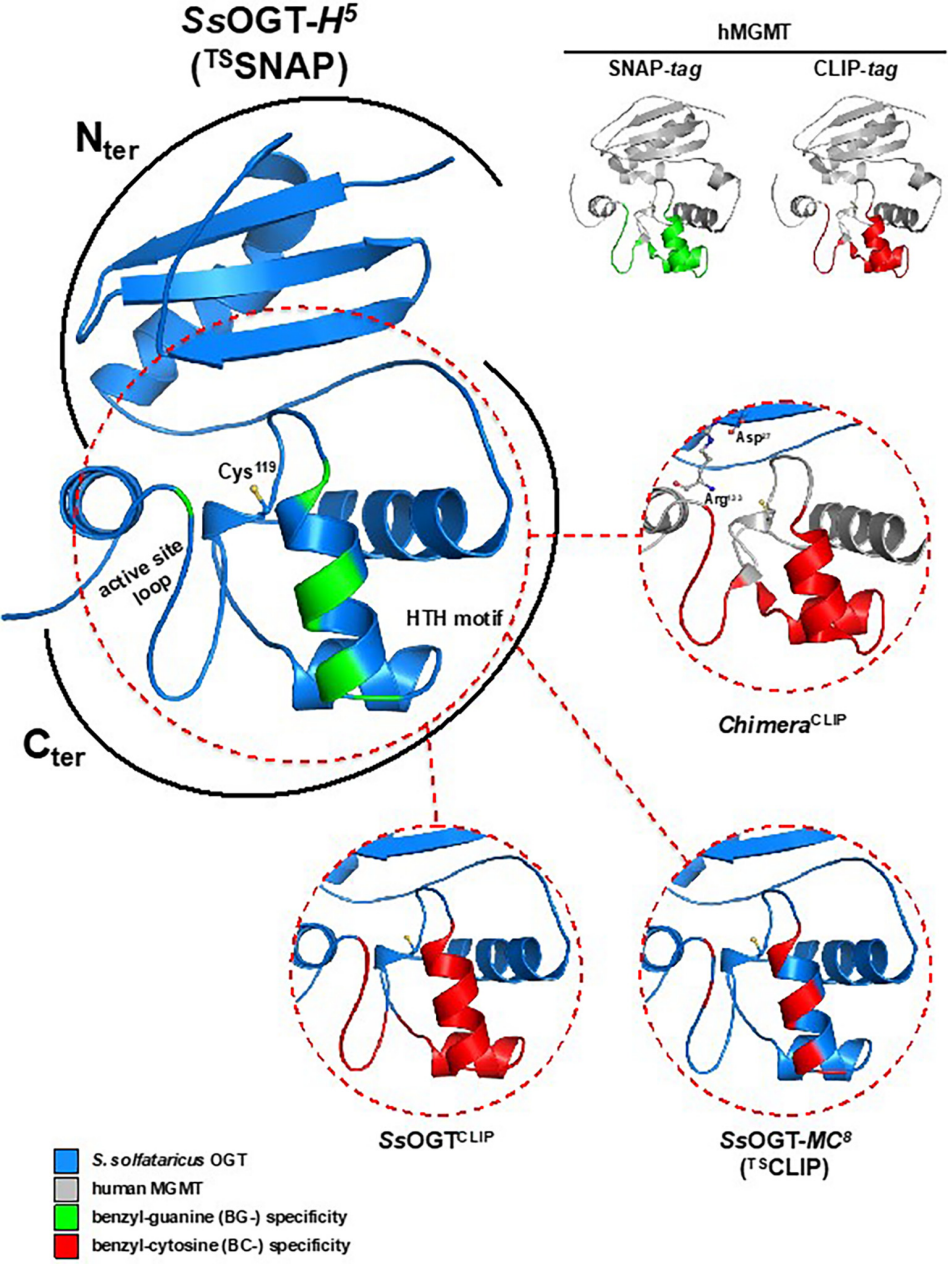
Commercial and thermostable SLPs. Cartoon representation of SLPs, with BG- and BC- substrate specificity coloured as indicated in the legend.

Despite the effort pushed on the hMGMT variants in order to increase their general stability [6], it is known that the SNAP-*tag* and the CLIP-*tag* have the limitation of being employable *in vitro* under moderate reaction conditions and *in vivo* in “mesophilic” model organisms characterized by a temperature optimum lower than 40 °C. Although these conditions cover most applications, these SLPs are not suitable for studies on (hyper)thermophilic organisms and, more generally, organisms that thrive in non-permissive environmental conditions (high pressure, extremes of pH, high ionic strength, etc.). To fill this gap, from 2012 on, Perugino and collaborators identified an *O^6^*-alkyl-guanine-DNA-alkyl-transferase activity in the hyperthermophilic archaea *Saccharolobus solfataricus*: as expected, this protein is involved in *in vivo* direct DNA repair by alkylating agent treatment [14]. The fruitful lesson from the SNAP-*tag*, where the aminoacidic residues involved in the recognition of the BG- derivatives were identified, led to the production by site-directed mutagenesis of a *S. solfataricus* OGT variant (defined *Ss*OGT-*H^5^*, Fig. 2 and Fig. S1), which has been fully characterized: it is unable to bind DNA but still retaining a high catalytic activity on the fluorescein BG-derivative (SNAP-Vista® Green, hereinafter BG-FL, Fig. S2) [14]. Furthermore, *Ss*OGT-*H^5^* revealed a strong thermostability comparable to the wild type and a general resistance to other chemical-physical denaturants [14]. Unlike hMGMT and relative variants, the OGT homologue from *S. solfataricus* is not sensitive to chelating agents [14], suggesting the absence of any structural Zinc ion: this was then confirmed by the 3D resolution of this archaeal protein [15]. All these premises constituted the starting point for exploiting this mutant as an innovative SLP in “thermophilic” contexts. Indeed, *Ss*OGT-*H^5^* was used as a protein tool genetically fused to a thermostable enzyme [16], as well as it underwent heterologous expression in thermophilic organisms [16], [17]. In particular, we purified at high yield and purity the *Ss*OGT-*H^5^*-lacS fusion protein by means of immobilized metal affinity chromatography (IMAC), as well as by incubating the *E. coli* cell free extract at high temperature (70 °C) in order to obtain a selective precipitation of *E. coli* proteome and recover the heterologously expressed thermostable protein in the soluble fraction [16]. Moreover, the presence of this thermostable SLP (hereinafter ^TS^SNAP) also allowed a fourfold increase the thermostability of the alpha-carbonic anhydrase from *Sulfurihydrogenibium yellowstonense* [18], [19]. The heterologous expression of the ^TS^SNAP was successfully achieved in the thermophilic bacterium *Thermus thermophilus* HB27^EC^, as well as in the hyperthermophilic archaea *Sulfolobus islandicus* [16], [17]. In both cases, these organisms were permeable to BG-FL, making possible the determination and the measure of the activity of ^TS^SNAP, demonstrating the correct folding at high temperatures in these organisms [16], [17].

By means of computational and structural approach, we have rationally driven the tuning of the substrate specificity of the ^TS^SNAP from BG- to BC-derivatives, in order to produce and fully characterize the first, to date, thermostable CLIP-*tag* (^TS^CLIP; Fig. 2 and Fig. S1). The structure-based analysis allowed us to detect the peculiar amino acids involved in substrate specificity for this class of enzymes, as well as to propose a general approach for the engineering of any *O^6^*-alkyl-guanine-DNA-alkyl-transferase turning it into a SNAP- and a CLIP-*tag* variant.

## 2 Results and discussion

### 2.1 Production of a thermostable CLIP-*tag* by rational design

#### 2.1.1 General considerations

Currently, several dozen *O^6^*-alkyl-guanine-DNA-alkyl-transferases (E.C: 2.1.1.63) have been fully characterized (see BRENDA web site, https://www.brenda-enzymes.org/enzyme.php?ecno=2.1.1.63; see UniProt web site, https://www.uniprot.org/uniprot/?query=2.1.1.63&sort=score) and more than forty 3D structures were present in Protein Data Bank (including 3D structures of variants, in complex with DNA or ligands, etc.), highlighting the molecular mechanisms of these proteins in the direct DNA repair and substrate specificity. Spanning from mesophilic to thermophilic environments, AGTs display a different primary structure, whereas all of them show a typical protein architecture, mainly consisting of two globular domains [8], [20]: a poorly conserved N_ter_ domain, whose function is not still well understood (likely involved in regulation, cooperative binding, and stability; [15], [21], [22]), and a highly conserved C_ter_ domain housing all the functional elements for protein activity; i.e., the helix-turn-helix motif (HTH) responsible for the DNA binding, the -V/IPCHRVV/I- amino acid consensus sequence including the catalytic cysteine, and the *active site loop* that is mainly involved in the substrate specificity and it is characterized by a conformational plasticity for which it is annotated as a structural element undergoing spatial rearrangement along the catalytic cycle (Fig. 2) [8], [21], [22], [23], [24], [25].

The 3D structure of *Ss*OGT revealed some peculiarities of this thermostable protein in the N_ter_ domain, as the presence of a S—S bridge (C29-C31), the absence of any structural Zinc ion, and the interconnection between the N_ter_ and the C_ter_ domain by the D27-R133 residues interaction [15]. Site directed mutagenesis targeting the above-mentioned residues demonstrated their role on thermal stability of this protein [15]. Based on these data, we explore a structure- and computational-based pipeline to design a thermostable OGT specifically active on BC-derivatives for biotechnological application which require an SLP with an orthogonal substrate recognition both for molecular *in vitro* studies and for *in vivo* multi-protein labelling.

Accordingly, the experimental workflow described below includes three key steps: *i*) primary sequence design; *ii*) *in-silico* structural analysis to select the molecular determinants of thermal stability and substrate specificity; *iii*) engineered protein purification and biochemical characterization.

#### 2.1.2 The Chimera^CLIP^ mutant

The molecular evolution approach leading to the production of the SNAP-*tag* and the CLIP-*tag* starting from hMGMT, clearly revealed that, as expected, the mutations for the enhancement of the activity towards BG-, as well as for the change in substrate specificity towards BC-derivatives mainly fall in the C_ter_ domain, in particular in the *H4* helix of the HTH motif and the *active site loop* (Fig. 2 and S1) [6], [12]. The first attempt to produce a thermostable OGT active on BC-derivatives was indeed the construction of a chimeric gene, by combining the N_ter_ domain (including the *connecting loop*) of *Ss*OGT with the C_ter_ domain of the CLIP-*tag*. Trying to assure enough stability to this chimera, the well-known D27-R133 ion-pair of *Ss*OGT [15] was introduced, by replacing a glycine residue in the CLIP-*tag* C_ter_ domain with an arginine residue (G160R mutation). This chimera (*Chimera^CLIP^*; Fig. 2 and Fig. S1) inside *E. coli* cells revealed a specific activity on CLIP-Cell™ TMR-Star (hereinafter BC-TMR; Fig. S2), demonstrating that the selectivity offered by the C_ter_ domain coming from the commercial *tag* was not influenced by the presence of a different N_ter_ domain from the *Ss*OGT (Fig. S3a). This very promising preliminary result, however, was hampered by the purification trials failure, probably due to the strong instability of the chimeric protein.

#### 2.1.3 The tailored gene expressing SsOGT^CLIP^

Afterwards, a synthetic gene of *Ss*OGT was produced, where its *H4* helix and the active site loop were substituted by the same structures from CLIP-*tag* (the so-defined “CLIP” region in Fig. S1), leading to the purification of *Ss*OGT^CLIP^ (Fig. 2 and Fig. S1).

**Fig. 3:**
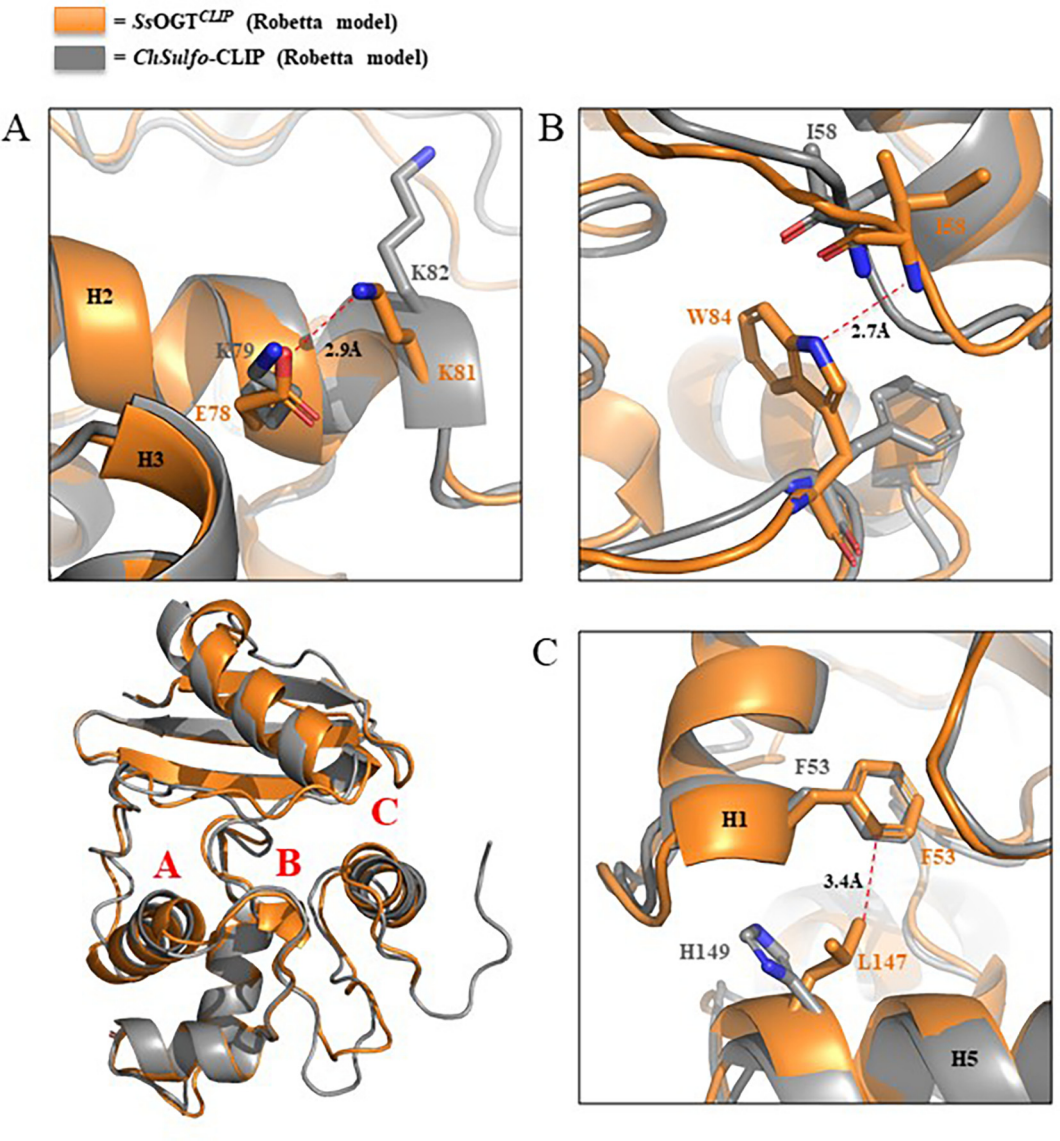
*Molecular contacts gained by SsOGT^CLIP^ with respect to Chimera^CLIP^.*Fig. 3a represents the ion pair between E78 and K81; Fig. 3b shows the N_ter_-to-C_ter_ contact based on H-bond of W84 with I58; Fig. 3c highlights the hydrophobic interaction between L147 and F53. Each panel is indicated with the corresponding letter (*in red*) in the cartoon representing the optimal superposition of *Ss*OGT^CLIP^ and *Chimera^CLIP^* predicted structures, depicted *in orange* and *grey*, respectively. (For interpretation of the references to colour in this figure legend, the reader is referred to the web version of this article.)

In order to rationalize the target protein design, we generated the predicted tertiary structure of *Ss*OGT^CLIP^ and Chimera^CLIP^ using Robetta (https://robetta.bakerlab.org/) server [30]: by optimal superposition of the predicted models with the experimental structure of *Ss*OGT (PDB ID: 4ZYE) [15], we were able to validate the potential improved stability of *Ss*OGT^CLIP^*,* mainly based on the introduction, among the others, of three aminoacidic substitutions belonging to the wild type *Ss*OGT, i.e., K78E, F84W and H147L. In particular, the mentioned mutations led to the instauration of an ion pair, a H-bonds and a hydrophobic contact, respectively (Fig. 3). Interestingly, two of the resulting contact are responsible for molecular bridge connecting the *N*-terminal to the C-terminal domain. Indeed, the side chain of W84 is involved in a H-bond with the carboxylic oxygen of the I58 main chain, on the other hand L147 of H5 produced a hydrophobic interaction with F53 that lies on *H1* helix.

Although the heterologous expression of this protein was quite low, a few amounts was purified and tested with BG- and BC- fluorescent derivatives. As shown in Fig. S3b, by a qualitative assay with fluorescent substrates, we revealed that ^TS^SNAP and *Ss*OGT^CLIP^ possess a strong specificity towards their relative substrates, and an undetectable activity with the orthogonal ones, even after a prolonged incubation (see 5.4 Section). Unfortunately, also in the case of *Ss*OGT^CLIP^, heat treatment above 50 °C resulted in a thermal denaturation and *in vitro* protein precipitation of this protein (data not shown). The insertion of mesophilic fragments from the CLIP-*tag* in the scaffold of the *Ss*OGT led to protein instability as in the heterologous expression as well as in the thermal denaturation. For these reasons, we decided to maintain the overall primary structure of *Ss*OGT as much as possible, in order to hit only few aminoacidic residues in the wild-type protein, trying to preserve the thermophilic feature of the designed construct. Since biochemical evidences were in good agreement with the comparative structural analysis, we applied again the computational approach to drive the design of the *Ss*OGT-*MC^8^* mutant.

#### 2.1.4 Biochemical characterization of the SsOGT-MC^8^ mutant

Detailed information from the *in-silico* studies on *Ss*OGT were then applied for the construction of a synthetic gene, in which eight aminoacidic residues in the wild type were substituted, in particular four of them (S100A, R102A, M106T, K110E) were kept as in the ^TS^SNAP enzyme [16], in order to avoid any DNA binding activity. Other substitutions (G105N, S109D, G130P, S132L) coincide with the aminoacidic residues typical of the CLIP-*tag* enzyme (Fig. S1) from which we just excluded the Y-to-E mutation on *H3* helix keeping the Y90 in the primary structure of *Ss*OGT-*MC^8^*, in order to maintain the active site loop anchored to the *H4* helix by establishing H-bond with Y90 (the structure-based rationale of such aminoacidic mutation is discussed below).

The expressed protein, the *Ss*OGT-*MC^8^* (Fig. 2) was easily purified from the *E. coli* cell free extract by affinity chromatography. The catalytic activity (in terms of second-order rate constant; [12], [13], [15], [16], [26]) of this *tag* was determined at the relative physiological temperature (65 °C): as the previously citated engineered variants, it exhibits activity on the BC-TMR substrate, whereas it was not possible to measure any activity on orthogonal BG-FL substrate, displaying a specular behaviour with respect to the ^TS^SNAP (Table 1), thus impeding in both cases to determine a BC/BG ratio value. On the other hand, purified CLIP-*tag* reacts only 10^2^ faster on BC-TMR than on BG-FL, partially in agreement with previous data [12], [26], whereas SNAP-*tag* is generally more specific on BG-derivatives (10^3^ faster; Table 1) [12], [26]. The residual reactivity of CLIP-*tag* towards its non-respective substrate, makes it advisable to first label the SNAP-*tag* to minimize cross-reactions [26].

**Table 1:** Catalytic activity of commercial and thermostable SLPs on BG- and BC-derivatives.

enzyme	T (°C)	*k*_BC_ (s^−1^ M^−1^)	*k*_BG_ (s^−1^ M^−1^)	Ref.
SNAP-*tag*	25	2.6 × 10^1^ (BC-FL)	2.8 × 10^4^ (BG-FL)	[12]
SNAP-*tag*	25	3.2 × 10^2^ (BC-TMR)	4.3 × 10^5^ (BG-TMR)	[26]
CLIP-*tag*	25	1.1 × 10^3^ (BC-FL)	1.0 × 10^1^ (BG-FL)	[12]
CLIP-*tag*	25	1.9 × 10^4^ (BC-TMR)	8.3 × 10^1^ (BG-TMR)	[26]

CLIP-*tag*	25	1.2 × 10^5^ (BC-TMR)	9.4 × 10^3^ (BG-FL)	this study
*Ss*OGT-*H^5^*	65	ND^a^ (BC-TMR)	2.0 × 10^4^ (BG-FL)	this study
*Ss*OGT-*MC^8^*	65	9.1 × 10^3^ (BC-TMR)	ND^a^ (BG-FL)	this study

^a^ ND = not determined.

An analysis of the cross-reactivity of thermostable SLPs was therefore performed by employing two different approaches. First, by using fluorescent substrates in competition with customised non-fluorescent nucleobases (BG-1 and BC-2, Fig. S2 and S4) [15], [27], IC_50_ values were determined. As shown in Table 2, all enzymes particularly prefer fluorescent substrates respect to our customised product (Fig. S5), leading to very high IC_50_ values, if compared to classical AGTs’ inhibitor, as BG- and Lomeguatrib [28]. Nevertheless, *Ss*OGT-*MC^8^* and CLIP-*tag* displayed an expected high orthogonal specificity, but this behaviour could be an effect of the substrate pairs used [26].

**Table 2:** Cross-reactivity of SLPs by competitive inhibition method (IC_50_), by using fluorescent derivatives as substrates and non-fluorescent ones as competitors.

enzyme	T (°C)	substrate	competitor	IC_50_ (μM)
CLIP-*tag*	25	BC-TMR	BG-1	310.2 ± 81.7
CLIP-*tag*	25	BC-TMR	BC-2	11.8 ± 3.9
*Ss*OGT-*H^5^*	65	BG-FL	BG-1	1.5 ± 0.3
*Ss*OGT-*H^5^*	65	BG-FL	BC-2	17.3 ± 16.4
*Ss*OGT-*MC^8^*	65	BC-TMR	BG-1	ND^a^
*Ss*OGT-*MC^8^*	65	BC-TMR	BC-2	127.6 ± 31.1

^a^ND = not determined.

**Fig. 4:**
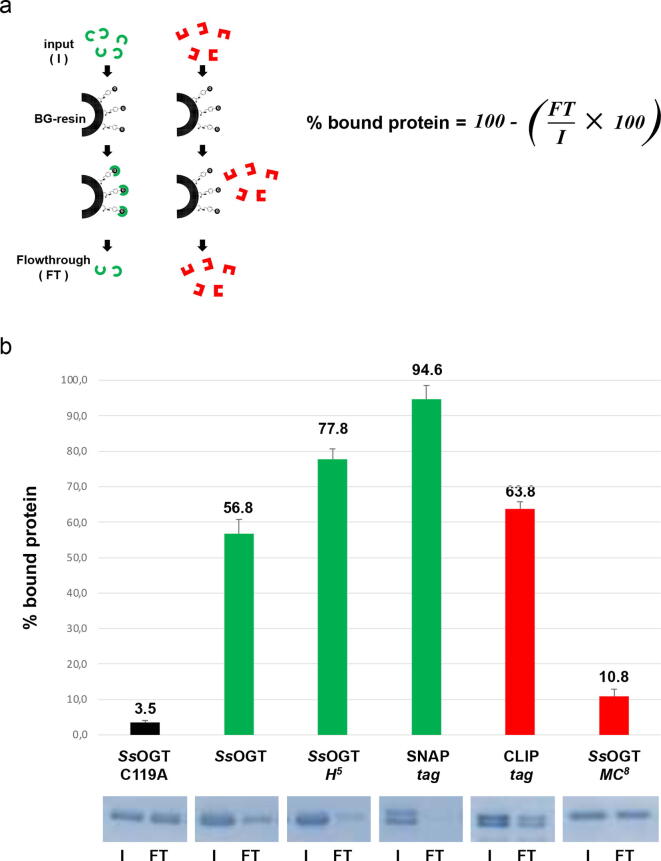
*Substrate specificity of CLIP-tag and SsOGT-MC^8^ by immobilization studies*. a) A fixed amount of protein (indicated as input, *I*) is added to a specific amount of a BG-resin: if the covalent reaction occurs (*green enzymes*), the resin captures the protein reducing the amount of non-bound protein (indicated as flow-through, *FT*), as measurable after SDS-PAGE analysis. On the contrary, orthogonal activity (*red enzymes*) does not allow the covalent binding of the protein on the resin. The value of the specific covalent binding is calculated by the equation shown in the figure; b) SDS-PAGE and histogram of the relative efficiency of binding of commercial and thermostable SLPs. Gels are an example of three independent experiments for each protein. (For interpretation of the references to colour in this figure legend, the reader is referred to the web version of this article.)

The second approach involved the utilization of a BG-conjugated agarose resin (SNAP-Capture Pull Down Resin, New England Biolabs), generally suitable for the SNAP-*tag* selective immobilization. As shown in Fig. 4a, the degree of the covalent protein immobilization depends from the total amount of protein (input, *I*) and the unbound protein in the flowthrough (*FT*) by following the equation in Fig. 4a. Only a specific activity on BG-derivatives (*green enzymes* in Fig. 4a) leads to the immobilization, increasing the value in term of percentage of bound protein on the resin.

Considering that this experiment was performed at room temperature (see Section 5.7), as expected, SNAP-*tag* is effectively the most active on the BG-resin, followed by the ^TS^SNAP and *Ss*OGT, confirming that the mutant is more active than the wild type at room temperature [16] (Fig. 4b). Because the inactive mutant *Ss*OGT C119A [15] does not interact with the BG- substrate on the resin, the covalent binding of BG-reactive SLPs is clearly from their specific activity (Fig. 4b).

The addition of BC-reactive SLPs on BG-resin (*red bars* in the histogram of Fig. 4b) resulted in a partial but not negligible immobilization of the CLIP-*tag*, whereas *Ss*OGT-*MC^8^* displayed a much lower protein immobilization (Fig. 4b), probably due to the low activity of this *protein-tag* at room temperature. All data clearly demonstrated that this variant of *Ss*OGT is effectively specific on BC-derivatives and could be used in combination with orthogonal BG-derivatives with a low risk of cross-reactivity.

Finally, we tested the thermal stability of *Ss*OGT-*MC^8^* by Differential Scan Fluorimetry (DSF), as described [15], [16], [29]. It is very important to underline that this technique is usually carried out from 20 °C to 95 °C at a ramping of 1 % (scan rate of 1 min/°C × cycle). Under these conditions, SNAP-*tag* showed a T_m_ over 65 °C, revealing an increased stability of ca. 17 °C respect to the wild type hMGMT counterpart [6]. In the case of thermostable OGTs from *Saccharolobus solfataricus* and *Pyrococcus furiosus*, they were not effectively denatured and it was not possible determine T_m_ values [15], [16], [29]. The experimental protocol was then opportunely modified by increasing to 5 min/°C × cycle and maintaining the temperature limits [15], [16], [29]. This prolonged heat treatment considerably reduced the T_m_ value of the SNAP-*tag* (Table 3 and Fig. S6), while the *Ss*OGT-*MC^8^* variant showed a stability to thermal denaturation almost identical to ^TS^SNAP [16], which is slightly lower than the *Ss*OGT wild type (80 °C) [16]. Indeed, the mutations introduced in *Ss*OGT in order to change the substrate specificity did not affect the thermal stability of the *Ss*OGT-*MC^8^* mutant, thus proposing it as a valid alternative of the CLIP-*tag* under high temperature conditions, both *in vivo* and *in vitro*.

**Table 3:** Protein stability of SLPs by the Differential Scan Fluorimetry method.

enzyme	rate (min/°C × cycle)	T_m_	note
SNAP-*tag*	5	51.32 ± 0.73	this study
*Ss*OGT-*H^5^*	5	73.87 ± 2.74	this study
*Ss*OGT-*MC^8^*	5	72.32 ± 1.03	this study

**Fig. 5:**
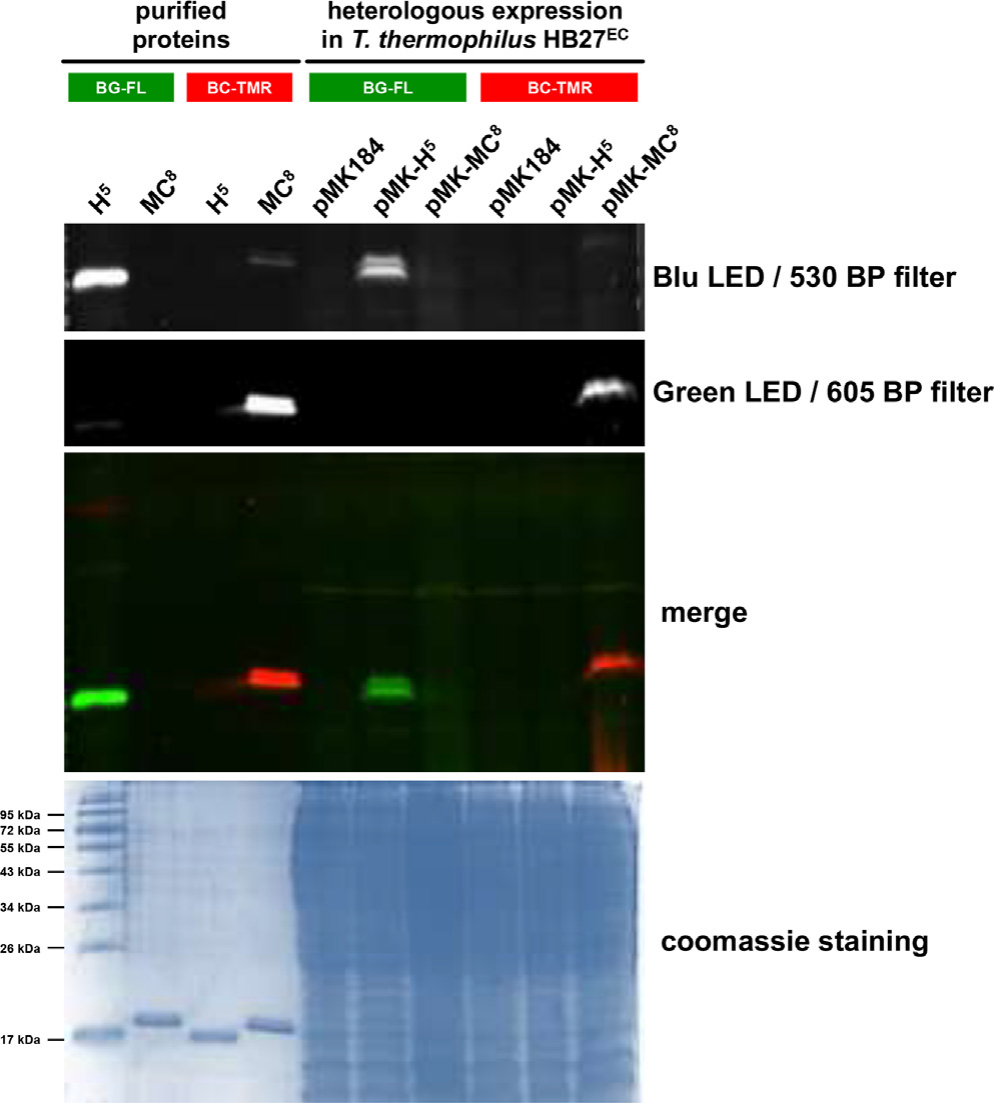
*In vivo heterologous expression of ^TS^SNAP and ^TS^CLIP in the thermophilic bacterium T. thermophilus HB27^EC^*. The *ogt*-*H^5^* and *ogt*-*MC^8^* genes were cloned into the pMK184 shuttle vector for their heterologous expression in HB27^EC^ strain. Overnight cultured cells were washed and incubated at 65 °C for 1 h in the presence of 1.0 μM fluorescent substrates (as indicated) and then subjected to SDS-PAGE (further details are described in the Section 5.4). The HB27^EC^/pMK184 strain was used as negative control, as well as 1 μg of each purified protein was used as positive control. In the *H^5^* lane was also loaded the protein marker.

*2.1.5. In vivo expression of ^TS^SNAP and SsOGT-MC^8^ in thermophilic bacteria.* The marked thermostability and the substrate specificity of this new variant have been tested in an *in vivo* experiment in the thermophilic bacterium *T. thermophilus* HB27^EC^, as described for ^TS^SNAP [16]. After an overnight culture at 65 °C, transformed cells with the empty pMK184 plasmid, as well as the constructs containing the *ogt-H^5^* and *ogt-MC^8^* genes, were centrifuged and resuspended in PBS 1 × buffer in the presence of fluorescent substrates. As seen for BG-FL [16], cells are also permeable to BC-TMR (Fig. 5), suggesting that also this substrate can be utilised in this model organism. Although the protein expression is far from being optimised, a specific fluorescent signal is only present where the *tags* with the own substrate are incubated (1 h at 65 °C), whereas the reaction with the orthogonal one did not reveal any fluorescent signal, reproducing the identical *in vitro* results employing purified proteins (Fig. 5). The presence of a specific BC- activity from the heterologous expression of the *ogt-MC^8^* gene clearly demonstrated that this SLP is correctly folded and, as the ^TS^SNAP, could be employed in thermophilic model organisms as ^TS^CLIP.

#### 2.1.5 The 3D structure of ^TS^CLIP

The presented crystallographic studies allow us to obtain the first structural model of a SLP with substrate specificity towards the BC-substrates (PDB ID: 8AES). ^TS^CLIP produced crystals that diffracted at 2.8 Å of resolution (Table 4) and thanks to the high-quality electron density map we were able to assign the orientation of all the amino acids along their sidechains, building the structural model of *Ss*OGT-*MC^8^* that shows the typical folding architecture of all AGTs, consisting of two globular domains connected by a long loop. Since the sequence is composed for the 95 % of the aminoacidic residues of *Ss*OGT, the engineered construct maintains the overall molecular architecture of *Ss*OGT including the determinants of the protein stability discussed below. In particular, the N_ter_ domain (a.a. 1–53) folds into an anti-parallel β-sheet, connected to a conserved α-helix (*H1*) by a random-coiled region; that is further stabilized by the peculiar disulphide bridge established between the C29 and C31. As mentioned above, the main modifications with respect to wild type *Ss*OGT occurred on the C-terminal domain, as it is directly involved in substrate recognition; indeed, it houses the catalytic C119 residue within the conserved -PCHR- signature, the “inactivated” HTH motif, responsible in wild type protein for the DNA minor groove binding, the active site loop and the ligand-binding pocket composed of the ‘asparagine hinge’ and *H4* helix.

**Table 4:** Data collection and refinement statistics.

dataset	*SsOGT-MC^8^*
*Data collection*	
Wavelength (Å)	0.9184
Resolution range (Å)	47.38–2.8 (2.9–2.8)
*Unit cell constants*	
a (Å)	100.835
b (Å)	119.435
c (Å)	180.237
α, β, γ (°)	90
Space group	P 21 21 21
N° of molecules in asu^a^	10
N° of reflections	185,454
N° of unique reflections	53,272 (7791)
R_pim_ (%)	8.1
R_merge_ (%)	13.8
R_meas_ (%)	16.2
I/σ	7.1 (2.2)
Completeness (%)	98.4 (99.3)
CC1/2	0.988 (0.562)
Multiplicity	3.5
*Refinement*	
R-factor (%)	19.6
R-free (%)^b^	25.3
Number of non-hydrogen atoms	12,153
macromolecules	12,086
ligands	0
solvent	67
Average B-factor (Å^2^)	54
macromolecules	54.48
solvent	46.69
Average RMSD^c^	
bond (Å)	0.009
angle (°)	1.18
*Ramachandran statistics*	
Residues (%)	
in most favoured regions	92.34
in additional allowed regions	7.06
in disallowed regions	0.60
Clashscore	9.51
Rotamer outliers (%)	4.58

By optimal superposition of ^TS^CLIP with the wild type protein, we observed the repositioning of discrete regions in ^TS^CLIP, in particular the active site loop moves of 2.6 Å from the central core of the protein towards the bulk solvent; it could be referred to the simultaneous presence of P130 that imposes rigidity and L132 that is characterized by higher flexibility (B-factor = 57.35) compared to the wild type serine (B-factor = 26.27). As consequence of such conformational change, we notice that R133 side chain shifts towards *H4* inducing a restriction of the upper part of the active site pocket. However, in the new conformation R133 still maintains the salt bridge with D27, whose importance for protein stability has been described (Fig. S7) [15], [25], [30].

The crystallographic analysis of ^TS^CLIP allowed us to determine the molecular contacts responsible for the achieved stability of such protein compared to *Ss*OGT*^CLIP^*, confirming the reliability of protein engineering process performed based of the predicted structure [31].

**Fig. 6:**
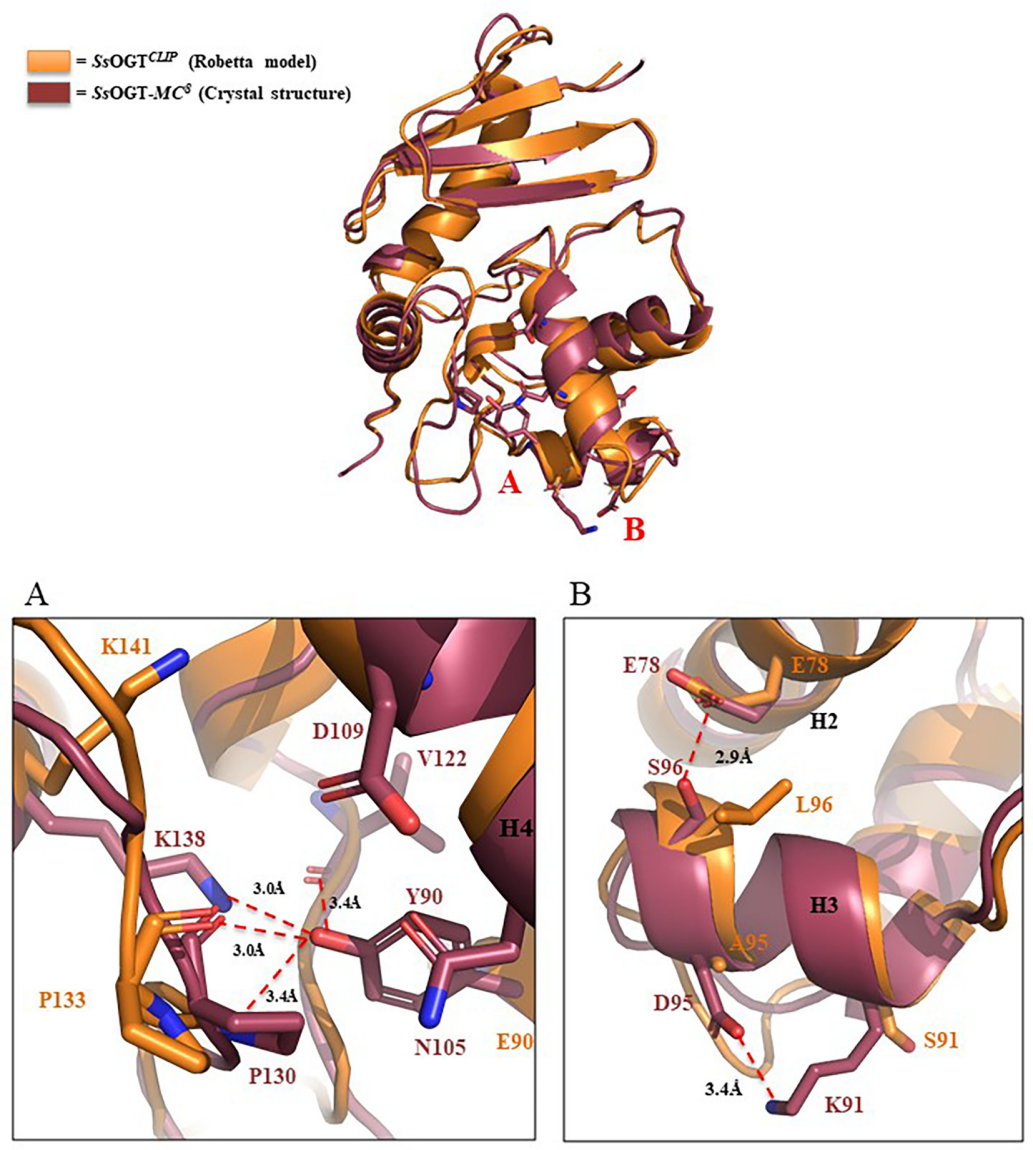
*Molecular contacts gained by ^TS^CLIP with respect to SsOGT^CLIP^.*Fig. 6a is a zoom-in of the network of contacts coordinated by Y90 in which we highlighted the potential H-bond between Y90 and the main chain of P130, V122 and the side chain of K138 respectively. Fig. 6b represents the *H3* intra-helix ion pair between D95 and K91 and the H-bond established by S96 and E78; the latter substitutes the intra-helix contact of *Ss*OGT^CLIP^ (*orange*) with an inter-helix bond in ^TS^CLIP (*raspberry*). (For interpretation of the references to colour in this figure legend, the reader is referred to the web version of this article.)

As observed in Fig. 6, ^TS^CLIP gained an intrahelical ion bond between D95 and K91, and a second H-bond between E78 and S96, connecting *H2* and *H3* helices, both bettering the overall stability of the enzyme; the main chain I128-K138 H-bond was restored too, as occurred in *Ss*OGT, anchoring the *H5* helix to the ligand binding cavity wall, opposing the one constituted by the HTH. In addition, the substitution of a glutamic acid with a tyrosine (E90Y) with respect to *Ss*OGT*^CLIP^* has a huge relevance, for the fact that Y90 is involved in the coordination of a H-bond-based network with V122, P130 main chain and K138: all these residues are located on the ligand entry site, making these interactions important functional elements for the selectivity towards BC-TMR substrates.

**Fig. 7:**
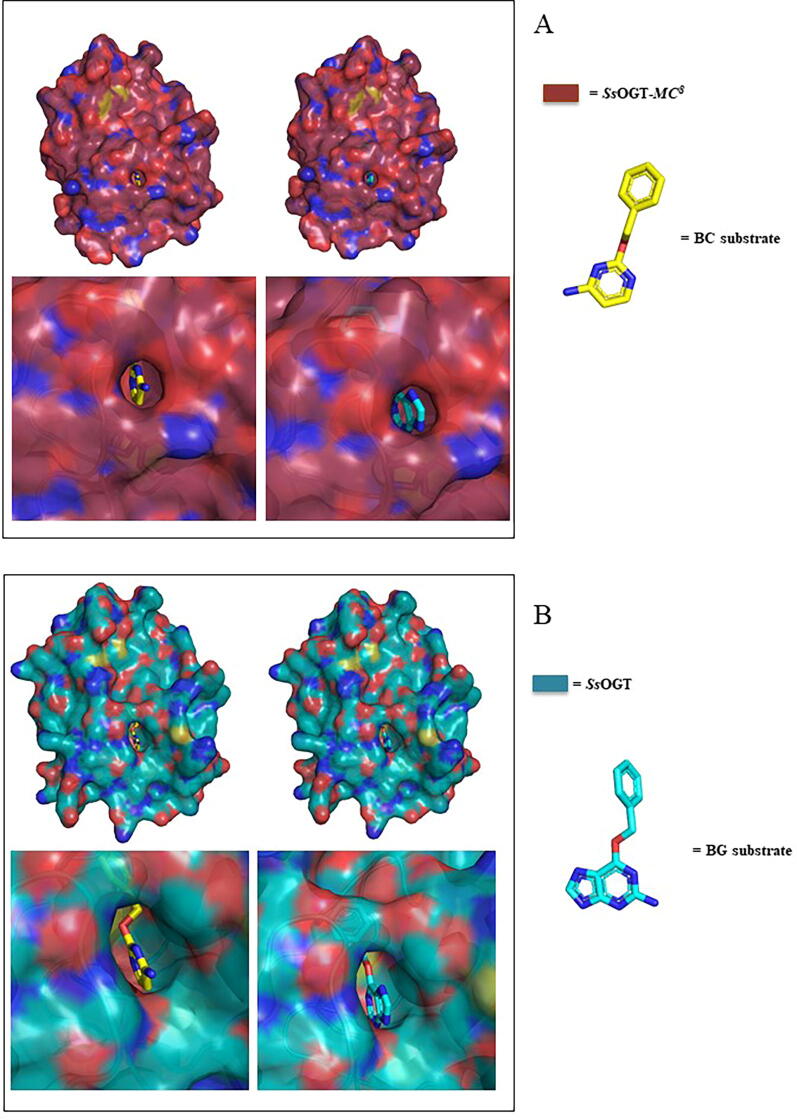
*Surface representation of crystal structures of SsOGT and ^TS^CLIP bound to the BC- and BG-derivatives.*Fig. 7a refers to ^TS^CLIP (*raspberry*) bound to BC (*on the left*) and BG (*on the right*) with a zoom on the active site entrance showing the difficult accessibility of BG imidazole moiety; Fig. 7b refers to the same comparison performed on wild type *Ss*OGT (*cyan*) (representative of ^TS^SNAP) highlighting the wider active site gate of this construct. (For interpretation of the references to colour in this figure legend, the reader is referred to the web version of this article.)

Being the first crystal structure of a SLP with binding activity on BC-derivatives we were able to depict the role of each selected mutations (G105N, S109D, G130P, S132L, Fig. S1) introduced to guarantee the substrate specificity, performing also a comparative analysis with the mutated residues included in CLIP-*tag* [12]. In particular, N105 and D109 on *H4* helix, introduced for the coordination of cytosine moiety, and P130 on the opposite surface contribute to narrow the accessibility to the substrate binding pocket by steric hindrance (Fig. 7).

In fact, by comparing the active site gate of the wild type *Ss*OGT with those of ^TS^CLIP (both crystallographic structures) in complex with a BC molecule optimally superposed to the BG ligand of the SNAP-BG complex (PDB ID: 3KZZ) it is evident that *Ss*OGT-*MC^8^* active site entry is shaped to host the BC moiety, while it clashes with the BG compound. Considering the steric contribution, the P130 plays a key role (Fig. 7) in re-shaping the active pocket entry, also because its conformation is stabilized by H-bond with Y90. Along playing a relevant role in the interaction with P130, Y90 (Y114E mutation in CLIP-*tag*) is the only residue that has not been modified by following the CLIP-*tag* generation protocol [12].

Johnsson and co-workers speculated on the possible role of such tyrosine in the formation of a hydrogen bond with the N_3_ of BG to stabilize the developing negative charge on the leaving group guanine; consequently, they decided to mutate it into glutamic acid to abolish BG binding activity [12], [23]. On the contrary, ^TS^CLIP is characterized by a higher substrate specificity also in presence of such tyrosine. Differently from CLIP-*tag*, in ^TS^CLIP the selectivity has been achieved also by steric hindrance, obtained as result of the inserted mutations, that allow the binding of BC- instead of BG- derivatives.

This observation is further confirmed by the biochemical investigation in which it was not possible to determine the kinetic of binding of BG due to its complete exclusion from the active site pocket.

## 3 Conclusions and perspectives

This work, resulting from the lesson of the molecular evolution performed on the hMGMT, is the demonstration that the knowledge acquired by the group of prof. Johnsson led to the identification of residues involved in the substrate specificity of this enzyme [12]. Since the AGTs are evolutionary conserved [14], this information could become a tool to apply mutations rapidly and unequivocally to all known AGTs, in order to develop new engineered SLPs. The only limitation of this approach is that the identified AGTs must be sensitive to benzyl-guanine inhibition (note that *E. coli* AdaC is not inhibited by BG, so it is not active on BG-derived substrates [32], [33]). As shown in Fig. S8, the “SNAP-*tag* technology” could potentially be used in each model organism: if the chemical-physical growth conditions are compatible with commercial SNAP- and CLIP-*tag*, it is advisable to exploit them, given their high catalytic activity on BG- and BC-derived substrates. In other cases, it is possible to search for a specific BG- activity using fluorescent substrates and, starting from the acquired knowledge, proceed to the identification and appropriately the modification of the endogenous AGT, in order to produce homologous SNAP- and CLIP-*tag*, whose behaviours are compatible with the *in vivo* conditions of the organism used.

Using this methodology and starting from an archaeal OGT, which is stable at high temperatures and to the most common denaturing agents [14], we engineered it obtaining a pair of thermostable SLPs (^TS^SNAP and, for the first time, ^TS^CLIP). These new *protein-tags* could allow an expansion of this technology in (hyper)thermophilic organisms and in all non-permissive reaction conditions for commercial SLPs.

## 4 Experimental Section/Methods

### 4.1 General experimental procedures

IR spectra were recorded on an Avatar 370 FT-IR Techno-Nicolet apparatus. 1H (400 MHz) and13C (100 MHz) NMR spectra were measured on Bruker Avance 400 MHz spectrometer or on a Bruker Avance 500 MHz. Chemical shifts were referenced to the residual solvent signal (CDCl_3_: δH = 7.25, δC = 77.0, CD_3_OD: δH = 3.34, δC = 49.0, DMSO: δH = 2.50, δC = 39.5, (CO(CD_3_)_2_: δH = 2.05, δC = 206.7, 29.9). Reactions were monitored by thin-layer chromatography (TLC) on Merck 60 F254 (0.25 mm) plates, visualized by staining with 5 % H_2_SO_4_ in EtOH and heating. Organic phases were dried with Na_2_SO_4_ before evaporation. Chemical reagents and solvents were purchased from Sigma-Aldrich, TCI Europe or Fluorochem and were used without further purification unless stated otherwise. Petroleum ether with boiling point of 40–60 °C was used. Silica gel 60 (70–230 mesh) was used for gravity column chromatography (GCC).

#### 4.1.1 Synthesis of BG-1

to a stirred solution of BG-NH_2_ (900 mg, 3.31 mmol, 1 eq. mol) in AN/DMF dry 1:1 (10 mL), 6-azidoesanoic acid (804 mg, 3.981 mmol, 1.2 eq. mol), triethylamine (1.85 mL, 13.27 mmol, 4 eq. mol) and T3P (sol. 50 % in EtOAc, 2.45 mL, 3.98 mmol, 1.2 eq. mol) were sequentially added. The reaction was stirred under N_2_ at 40 °C overnight, quenched with a saturated solution of NH_4_Cl and extracted with EtOAc. The organic phases were washed with brine, dry over sodium sulfate and evaporated under reduced pressure. The crude was purified by gravity column chromatography on silica gel (gradient PE-EtOAc from 3:7 to EtOAc-MeOH 85:15) to afford BG-1 as a white solid (500 mg, 35 % yield). ^1^H NMR (400 MHz, CD_3_OD) *δ* 7.85 (brs, 1H), 7.50 (d, *J* = 6.2 Hz, 2H), 7.31 (d, *J* = 6.2 Hz, 2H), 5.54 (s, 2H), 4.38 (s, 2H), 3.28 (t, *J* = 6.7 Hz, 2H), 2.26 (t, *J* = 7.3 Hz, 2H), 1.63 (m, 4H), 1.41 (m, 2H). HRESIMS *m*/*z* [M + H]^+^ 410.20480 (calcd for C_19_H_24_N_9_O_2_, 410.20475).

#### 4.1.2 Synthesis of BC-2

to a stirred solution of BC-NH_2_ (140 mg, 0.61 mmol, 1 eq. mol) in AN/DMF dry 1:1 (4 mL), 6-azidoesanoic acid (116 mg, 0.73 mmol, 1.2 eq. mol), triethylamine (333 μL, 2.44 mmol, 4 eq. mol) and T3P (sol. 50 % in EtOAc, 447 μL, 0.73 mmol, 1.2 eq. mol) were sequentially added. The reaction was stirred under N_2_ at 40 °C overnight, quenched with a saturated solution of NH_4_Cl and extracted with EtOAc. The organic phases were washed with brine, dry over sodium sulfate and evaporated under reduced pressure. The crude was purified by gravity column chromatography on silica gel (gradient PE-EtOAc from 3:7 to EtOAc-MeOH 85:15) to afford BC-2 as a pale-yellow solid (102 mg, 40 % yield). ^1^H NMR (400 MHz, CO(CD3)2) *δ* 7.97 (brs, 1H), 7.92 (d, *J* = 6.7 Hz, 2H), 7.40 (d, *J* = 6.6 Hz, 2H), 7.29 (d, *J* = 6.6 Hz, 2H), 6.20 (d, *J* = 5.7 Hz, 1H), 5.29 (s, 2H), 4.39 (d, J = 5.9, 2H), 3.33 (t, *J* = 6.8 Hz, 2H), 2.25 (t, *J* = 7.3 Hz, 2H), 1.63 (m, 4H), 1.42 (m, 2H). HRESIMS *m*/*z* [M + H]^+^ 370.19834 (calcd for C_18_H_24_N_7_O_2_, 370.19860).

### 4.2 DNA constructs

To obtain the *E. coli* expression vectors, similar procedures were applied for all the proteins. In particular, pQE-*CLIP-tag* and pQE-*ogtMC^8^* plasmids were used as templates to amplify the relative genes, by using Nco-NZY-Fwd / QE-Rev oligonucleotides pairs (5′-AGGAGATATACCATGGCACACCATCACCATCACCATACGG-3′ / 5′-CATTACTGGAT CTATCAACAGGAG-3′). Subsequently, the amplified fragments and the pHTP1 (NZYTech, Portugal) recipient vector were digested with Nco I and Hind III restriction enzymes and ligated. Then, the resulting ligation mixture was used to transform *E. coli* DH5α competent cells and positive colonies were confirmed by DNA sequence analyses. Whereas pQE-*ogtH^5^* was cloned as previously described [14]. In addition, to reach the final vector for the heterologous expression of *Ss*OGT-*MC^8^* mutant in *T. thermophilus* HB27^EC^ strain [34], pQE-*MC^8^* plasmid was first digested with *Bam*HI and *Hin*dIII enzymes to recover the *MC^8^* gene fragment, which was then subcloned in the pMK-*ogtH^5^* [16].

### 4.3 Protein expression and purification

^TS^SNAP was purified as previously described [16]. CLIP-*tag* and *Ss*OGT-*MC^8^* proteins were expressed in *E. coli* BL21 Rosetta2 (DE3) cells, grown at 37 °C in Lysogeny Broth (LB) medium supplemented with 50 mg/L kanamycin and 30 mg/L chloramphenicol [35], [36], [37]. The protein expression was induced with 1 mM isopropyl-thio-β-d-galactoside (IPTG) when an OD_600nm_ of 0.5–0.6 was reached. The biomass was collected and resuspended 1:3 (w/v) in purification buffer A (50 mM phosphate, 300 mM NaCl; pH 8.0) supplemented with 1 % Triton X-100 and stored overnight at −20 °C. Subsequently, it was treated in ice with lysozyme and DNAse for 60 min and sonicated as described [14]. After a centrifugation of 30 min at 60,000 × *g*, the cell extract was recovered and applied to a Protino Ni-NTA Column 1 mL (Macherey-Nagel) for His_6_-tag affinity chromatography, accordingly to the procedure previously described [27]. The eluted protein fractions were collected, dialysed against phosphate buffered saline (PBS 1×, 20 mM phosphate buffer, NaCl 150 mM, pH 7.3) and confirmed by SDS-PAGE analysis. To test the activity of purified proteins, 5 μM of enzyme was incubated with 10 μM of the relative substrate BG-FL and BC-TMR (New England Biolabs, USA) in 1 × Fluo Reaction Buffer (50 mM phosphate, 0.1 M NaCl, 1.0 mM DTT, pH 6.5) at 25 °C for CLIP-*tag* and 65 °C for *Ss*OGT-H^5^ and *Ss*OGT-*MC^8^*. After stopped the reaction in Laemmli buffer 1 × (formamide 95 %; EDTA 20 mM; bromophenol 0.05 %), samples were loaded on SDS-PAGE gel and analysed by *gel-imaging* technique on a VersaDoc 4000™ system (Bio-Rad) as previously reported [14], [15], [22].

### 4.4 *In vivo* fluorescent assay

For the *in vivo* assay, *T. thermophilus* HB27^EC^ cells transformed with pMK184 (used as control), pMK-*ogtH^5^* and pMK-*ogtMC^8^* plasmids were grown at 65 °C in SC selective medium (tryptone 8 g/L, yeast extract 4 g/L, NaCl 3 g/L, in mineral water pH 7.5) supplemented with 30 mg/L kanamycin as late as stationary phase (OD_600nm_ > 1.5) [16], [38]. Cell pellets from 1 mL were resuspended in 0.1 mL of SC medium in presence of 3 μM of BG-FL and BC-TMR fluorescent substrates and incubated at 65 °C for 1 h. After the reaction, cells were first washed twice with 1 mL of SC medium, then denatured for 15 min at 100 °C by adding a Laemmli buffer 1 × and directly loaded on SDS-PAGE.

### 4.5 Substrate specificity assay by competitive IC_50_ inhibition method

The substrate specificity of CLIP-*tag*, *Ss*OGT-*H^5^* and *Ss*OGT-*MC^8^* proteins were evaluated on BG-N_3_ and BC-N_3_ substrates by the competitive inhibition assay performed as described [15], [27], [29]. By using fixed concentrations of the fluorescent BC-TMR substrate (5 μM) and enzymes (5 μM), an increasing concentration of guanine/cytosine-azide derivatives (0–1 mM) was added to the mixtures. The reactions were incubated for 60 min at 25 °C for CLIP-*tag* and 65 °C for *MC^8^* respectively and then stopped by adding Laemmli buffer 1 ×. Subsequently, the samples were loaded on SDS-PAGE and the fluorescent bands were measured by *gel-imaging* on a VersaDoc 4000™ system (Bio-Rad), by applying green LED/605 bandpass filter. Then, obtained data were plotted by IC_50_ equation [15], [25]. As control, 5 μM of *Ss*OGT-*H^5^* protein was incubated at 65 °C with 5 μM of the fluorescent substrate BG-FL in presence of an increasing amount of the BG/BC-azide derivatives (0–1 mM). In this case, fluorescent bands were visualized on VersaDoc 4000™ system (Bio-Rad), by applying a blue LED/530 bandpass filter.

### 4.6 Rate constants determination

5 μM of purified CLIP-*tag*, *Ss*OGT-H^5^ and *Ss*OGT-*MC^8^* proteins were incubated in PBS 1 × buffer with an excess of BG-FL/BC-TMR substrates (20 μM) at 25 °C and 65 °C respectively, accordingly to the method described [12], [27].^.^ Aliquots were taken at different times and the reactions were immediately stopped in 1 × Leammli Buffer. Finally, the samples were loaded on SDS-PAGE for the *gel-imaging* analysis and data were fitted to a *pseudo*-first-order reaction model using the GraFit 5.0 software package (Erithacus Software ltd.), and second-order rate constants *k* (in s^−1^ M^−1^) were obtained by dividing the pseudo-first-order constant by the concentration of substrate.

### 4.7 Capture and protein immobilization of SLPs

The SNAP-Capture Pull Down resin (New England Biolabs, binding capacity: 1 mg pure protein/mL of bed resin) 80 µL was washed with 1 × PBS and incubated with 120 µL of 1 mg / mL of protein (SNAP-*tag* / CLIP-*tag* / *H^5^* / *MC^8^* / C119A mutant). After one minute, 5 uL of supernatant (protein plus resin) was withdrawn and the rest was incubated for 16 h at room temperature. Afterwards, 5 μL of supernatant (protein plus resin) was withdrawn and all samples were first loaded on SDS-PAGE and then protein bands were determined by coomassie staining. Visible *gel-imaging* was used for the determination of intensity of each protein band.

### 4.8 Thermal stability analysis by using DSF method

The stability of the OGTs variants was analysed by the differential scan fluorimetry method (DSF) by following the protocol previously described [6], [15], [16], [25], [39]. Triplicates of each condition containing 25 μM of enzyme in PBS 1 × buffer and SYPRO Orange dye 1× (Invitrogen, USA) were subjected to a scan of 70 cycles at temperatures from 20 to 95 °C for 5 min/°C × cycle, in a CFX96 Touch Real-Time PCR (Bio-Rad). Relative fluorescence data were normalized to the maximum fluorescence value and plotted vs temperature. The resulting sigmoidal curves allowed the determination of the inflection points (T_m_ values) by fitting the Boltzmann equation [6], [15], [16], [25], [39].

### 4.9 Crystallographic studies

For crystallization trials, the protein was concentrated up to 7.5 mg/mL using Amicon Ultra-0.5 mL centrifugal filter devices (membrane MWCO = 10 kDa). Crystallization was performed by means of a robot-assisted (Oryx4; Douglas Instruments) sitting-drop-based spare-matrix strategy, using screen kits from Hampton Research and Qiagen, by the vapor diffusion method. Optimal *Ss*OGT-*MC^8^* crystals grew in two weeks at 20 °C in a drop obtained by mixing equal volumes of a protein and a reservoir solution containing 0.2 M tri-Potassium citrate and 20 % (w/v) PEG3350 in a final droplet volume of 2 µL, equilibrated against 50 μL of the reservoir solution. For X-ray data collection, crystals were cryoprotected in the precipitant solution supplemented with 12 % glycerol, mounted in a cryo-loop and flash frozen in liquid nitrogen for subsequent X-ray diffraction analysis. The crystals diffracted at 2.8 Å of resolution and data were collected at the ID30-B beamline at the European Synchrotron Radiation Facility (ESRF, Grenoble, France) equipped with a Pilatus PILATUS3 6 M 1000 µm Si sensor (Dectris) [40], at a wavelength of 1.008 Å. The diffraction data were indexed with XDS program [41], which assigned the crystal to the orthorhombic space-group *P*212121 with the following cell dimension: a = 100.83 Å, b = 119.43 Å, c = 180.24 Å, α = β = γ = 90°. This cell contained 10 molecules per asymmetric unit, with a corresponding solvent content of 56.9 % and a Matthews coefficient of 2.85. Data processing was carried out using the CCP4 program suite [42] and in particular the structure was solved by molecular replacement using the program Phaser of the PHENIX software suite [43]; the structure of the wild type *Ss*OGT protein was used as the search model (pdb: 4ZYE) and the overall sequence identity between the two proteins was 95 %. The resulting electron density map was good enough to allow iterative cycles of manual model building using Coot [44] and refinement using phenix.refine [43] (Table 4). The atomic coordinates and structure factors of the *Ss*OGT-*MC^8^* have been deposited in the Protein Data Bank (http://www.rcsb.org) under the accession code PDB ID: 8AES.

### 4.10 Tertiary structure mode

Tertiary structure model of *Chimera*^CLIP^, *Ss*OGT^CLIP^ and CLIP-*tag* were generated by Robetta (https://robetta.bakerlab.org/) servers [31]. The software PROCHECK from CCP4 program suite [44] was used to analyse the Ramachandran plot of generated models along with the bond angles and bond lengths of the protein structure.

## Declaration of Competing Interest

The authors declare that they have no known competing financial interests or personal relationships that could have appeared to influence the work reported in this paper.
