# Supplementary material for: First thermostable CLIP-tag by rational design applied to an archaeal O6-alkyl-guanine-DNA-alkyl-transferase
Source: Comput Struct Biotechnol J. 2022 Sep 18;20:5275–86. doi: 10.1016/j.csbj.2022.09.015 (PMC9519396; doi:10.1016/j.csbj.2022.09.015)
Supplement: Supplementary data 1 [file mmc1.docx]

~~8~~ 9. SUPPORTING INFORMATION

**Fig. S1.** *Primary structure of commercial and thermostable SLPs, in comparison with the wild type hMGMT and SsOGT*. Aminoacid residues are coloured as indicated in the legend.

**Fig. S2.** *BG- and BC-derivative substrates used in this work*.

**Fig. S3.** *The* ~~Ch~~*~~Sulfo~~*~~-CLIP~~ *Chimera*^CLIP^ *and the SsOGT^CLIP^ proteins*. a) *fluorescence-imaging* of *E. coli* heterologous expression of ~~Ch~~*~~Sulfo~~*~~-CLIP~~ *Chimera*^CLIP^. M, protein marker; P, purified ^TS^SNAP; C, IPTG-induced *E. coli* ABLE C/pQE-*ogt-H^5^*; NI and I, non-induced and induced *E. coli* cell; b) *fluorescence-imaging* and *coomassie staining* of an *in vitro* reaction of purified ^TS^SNAP and *Ss*OGT^CLIP^ in the presence of fluorescent substrates. Used filters parameters as in **Fig. 5**.

**
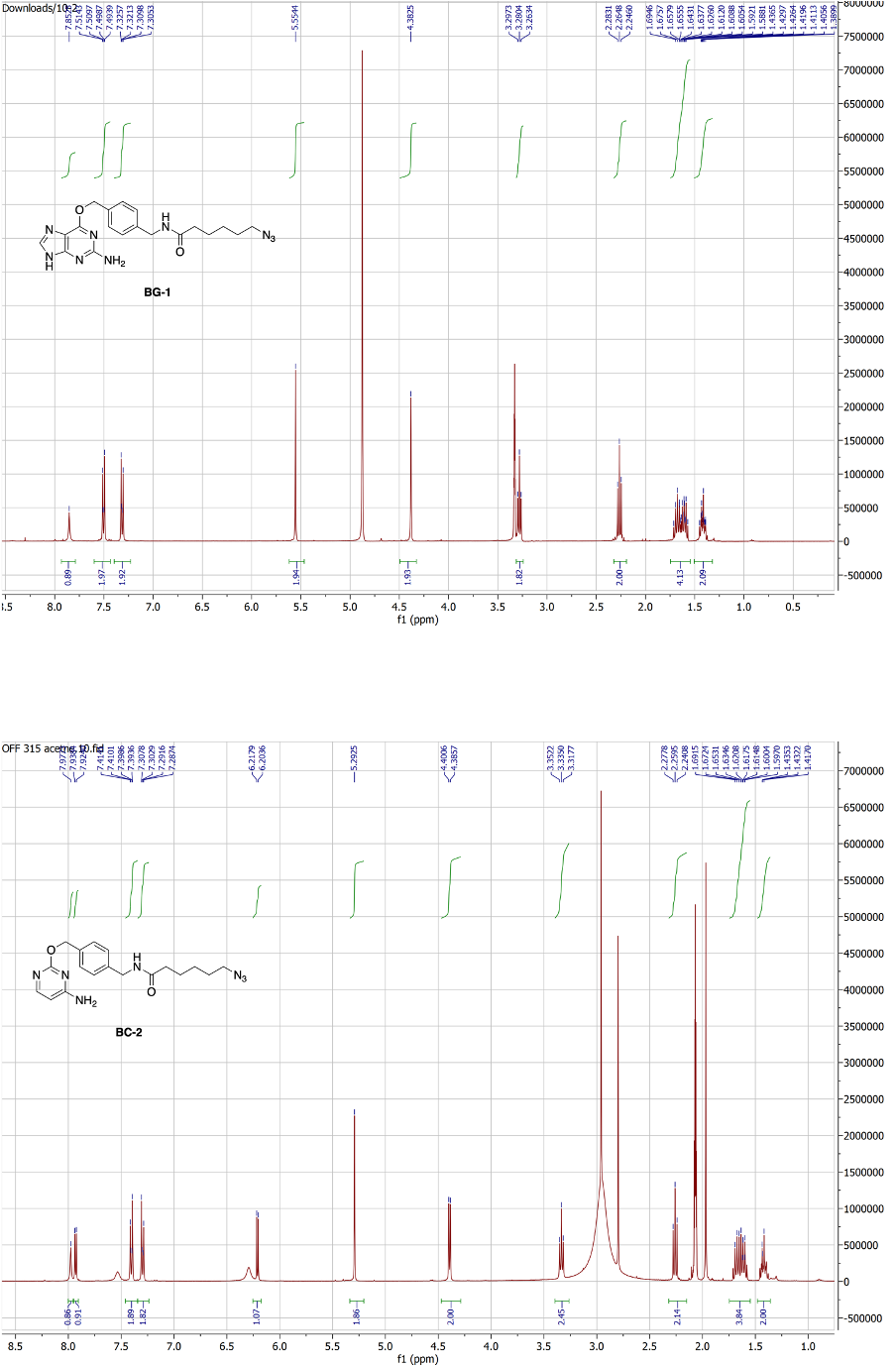
**

**Fig. S4.** *BG-1 and BC-2 spectroscopic characterization.*

**Fig. S5.** *Competitive inhibition studies of CLIP-tag and SsOGT-MC^8^*. An equimolar amount of protein and its fluorescent substrate (BC-TMR) was put in the presence of different amount of non-fluorescent competitors (BG-1 and BC-2), in order to evaluate the cross-reactivity of these enzymes. These gels are the example of three independent experiments. IC_50_ values are listed in the **Table 2**.

**Fig. S6.** *Differential Scan Fluorimetry plots of SNAP-tag and thermostable SLPs.*

**
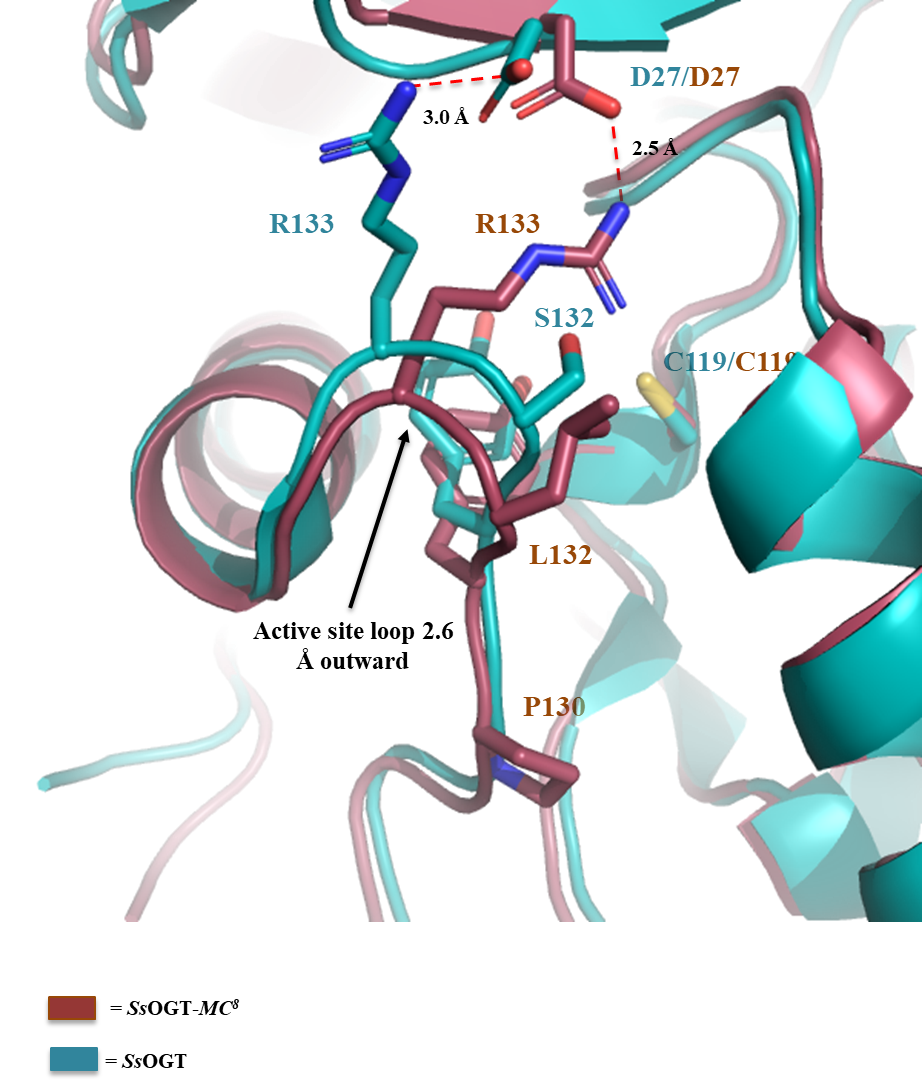
**

**Fig. S7.** *Active site loop repositioning observed in SsOGT-MC^8^ structure.* R133 moves toward the active site maintaining the ion pair with D27 and the active site loop shifts outward of 2.6 Å. *Ss*OGT-*MC^8^* is depicted *in raspberry* and *Ss*OGT wild type *in cyan*.

**Fig. S8.** *The “SNAP/CLIP workflow”*. The proposed general approach to obtain the “SNAP-*tag* technology” applied to all model organisms, included those whose growth conditions are not suitable for the employment of the commercial SNAP- and CLIP-*tag*.
